# Supplementary material for: MicroRNA-92a is a circadian modulator of neuronal excitability in Drosophila
Source: Nat Commun. 2017 Mar 9;8:14707. doi: 10.1038/ncomms14707 (PMC5347142; doi:10.1038/ncomms14707)
Supplement: Supplementary Information — Supplementary Figures and Supplementary Tables. [file ncomms14707-s1.pdf]

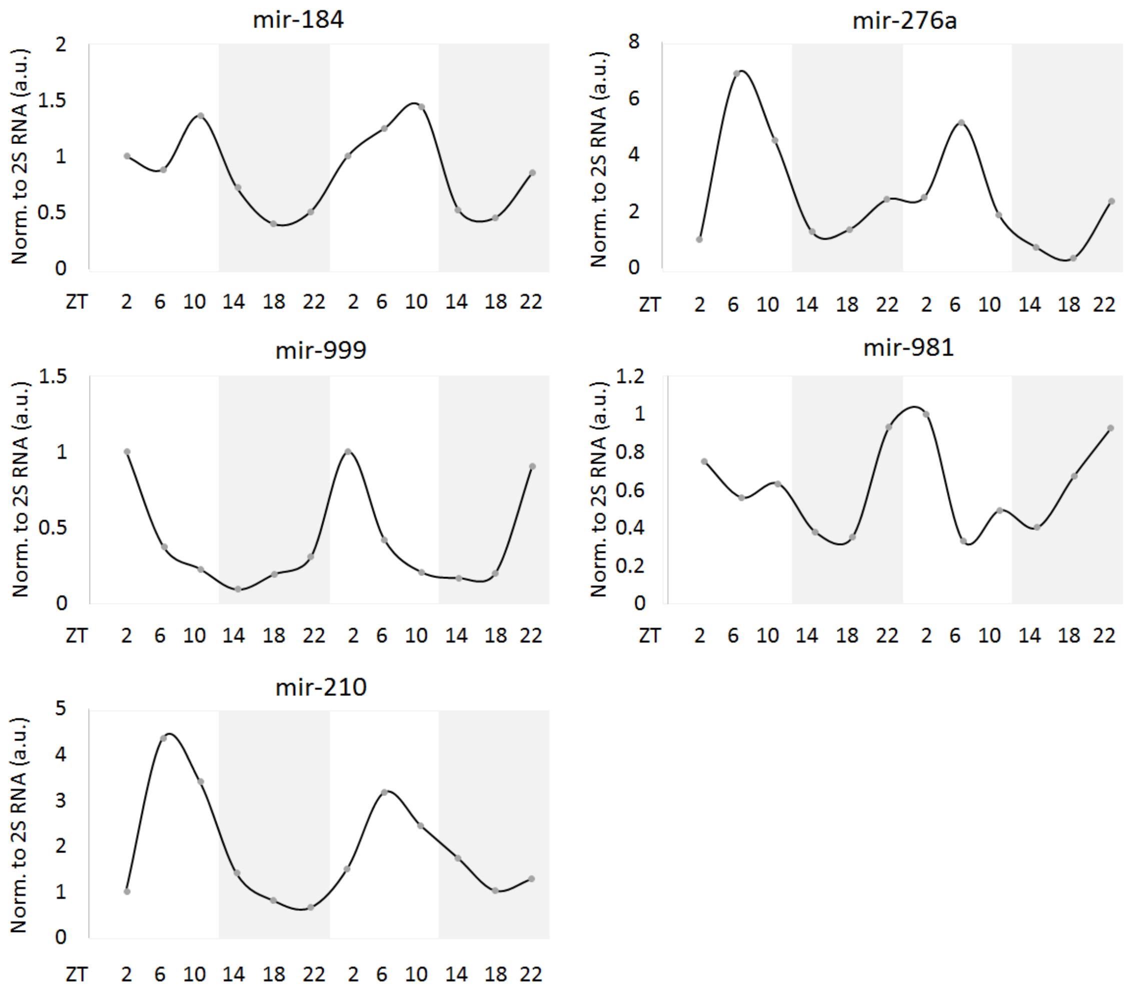

Supplementary Fig. 1 Expression levels of 5 miRNAs cycling in PDF cells under LD conditions. RT-qPCR quantification of miRNA levels in PDF cells entrained under LD cycles in WT flies (*PDF-GAL4;UAS-mCD8::GFP)*. miRNA expression levels are normalized to 2S rRNA. 2 biological replicates are concatenated to show cycling. a.u. represents artificial units.

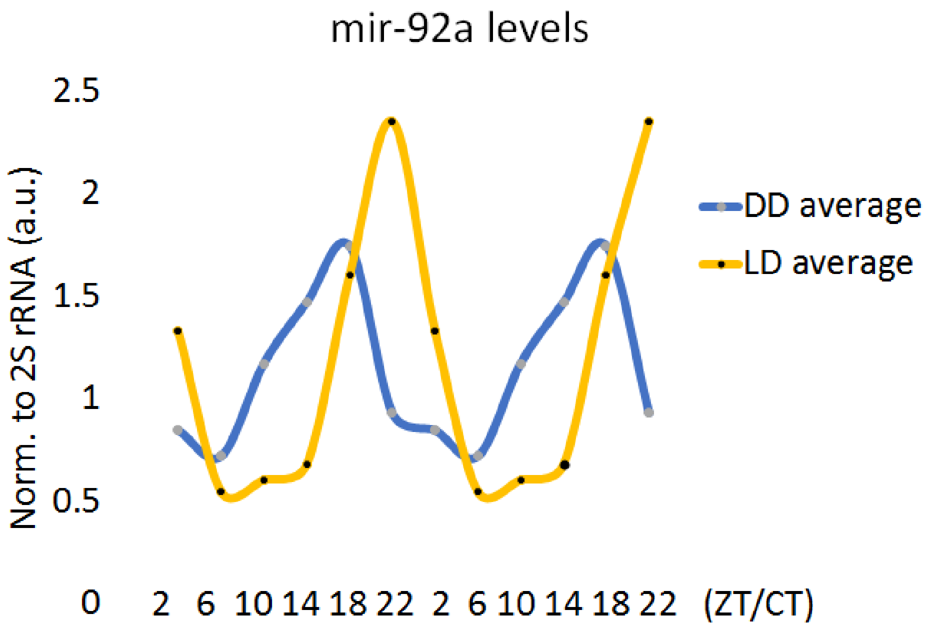

Supplementary Fig. 2 Expression levels of mir-92a under LD or DD conditions.

The average expression levels of 3 biological replicates under either LD or DD conditions in *PDF-GAL4;UAS-mCD8::GFP* flies are double plotted and superimposed to show the phase difference.

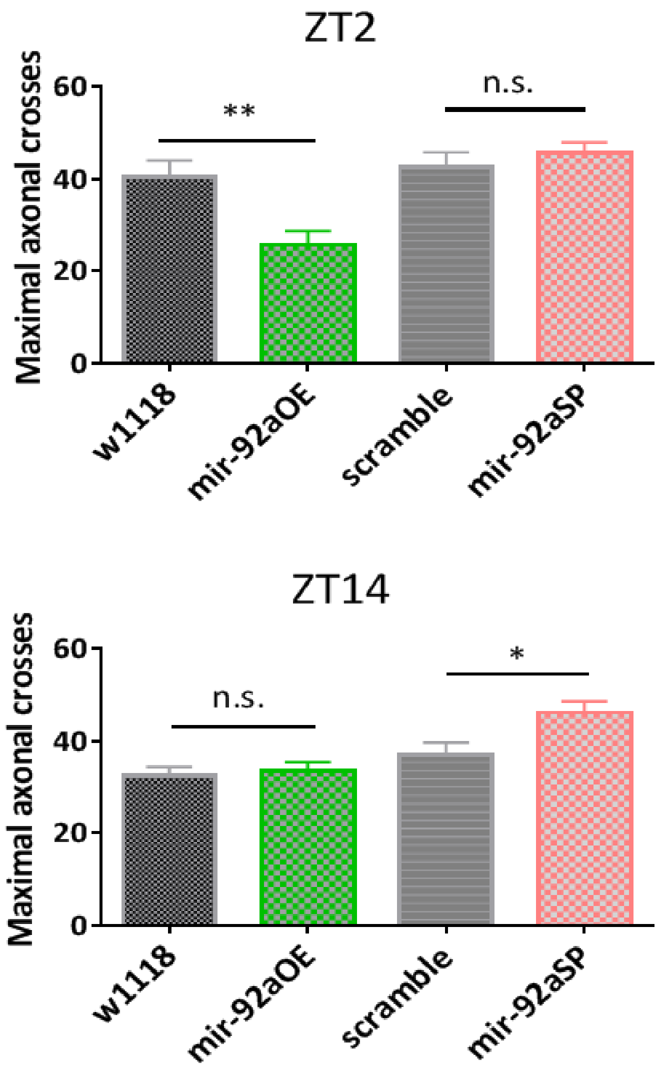

Supplementary Fig. 3 Statistical analysis of maximum axonal crosses quantification.

Axonal crosses were quantified with Sholl analysis (Fig. 2A). Maximum axonal crosses indicate the position where maximum amounts of intersections are found between axon branches of a projection and concentric circles in Sholl analysis. N = 14. Error bars represent ±SEM, n.s. indicates non-significant, *$P < 0.05$, **$P < 0.01$, one-way ANOVA.

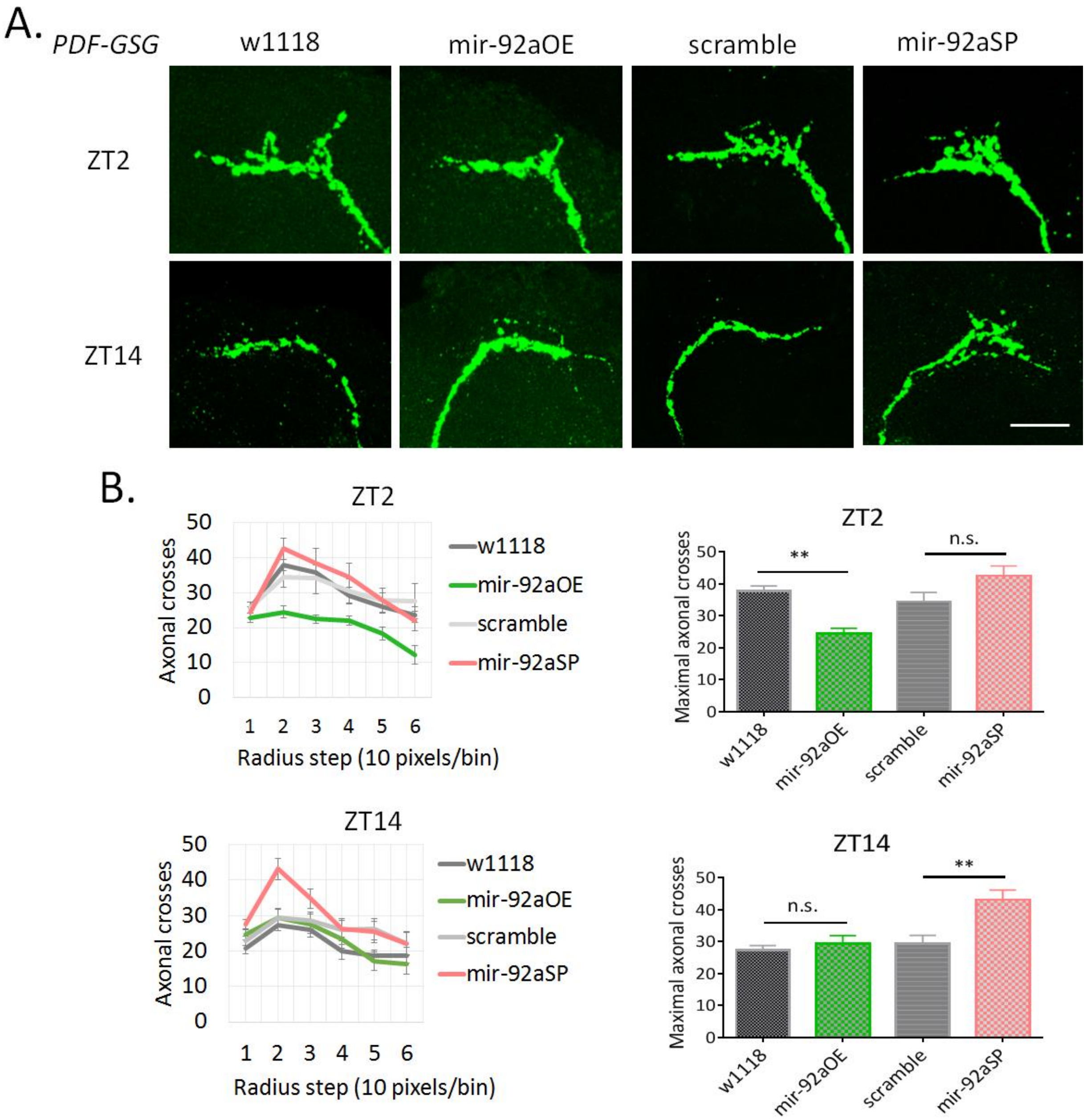

Supplementary Fig. 4 Adult-specific manipulation of mir-92a levels changes PDF cell fasciculation status.

Immunostaining of PDF cell projections with anti-PDF antibody at ZT2 or ZT14. mir-92aOE indicates *PDF-GSG;UAS-mCD8::GFP;UAS*-mir-92aOE flies and is compared to its corresponding control *PDF-GSG;UAS-mCD8::GFP*/+ (with w1118 background). mir-92aSP

indicates *PDF-GSG;UAS-mCD8::GFP;UAS*-mir-92aSP flies and is compared to its control, *PDF-GSG;UAS-mCD8::GFP;UAS*-scramble. Flies were fed on food containing 0.2 mg/mL Mifepristone (Sigma-Aldrich) for 1 week and entrained for at least 3 days under LD cycles prior to the assay.

(A) Representative images of PDF cell projections of the indicated genotype at the indicated time. Scale bar equals 25 µm.

(B) Quantification with Sholl analysis. Statistics were done on the points with maximum axonal crosses. N = 14. Error bars represent ±SEM, n.s. indicates non-significant, $^{**}P < 0.01$, one-way ANOVA.

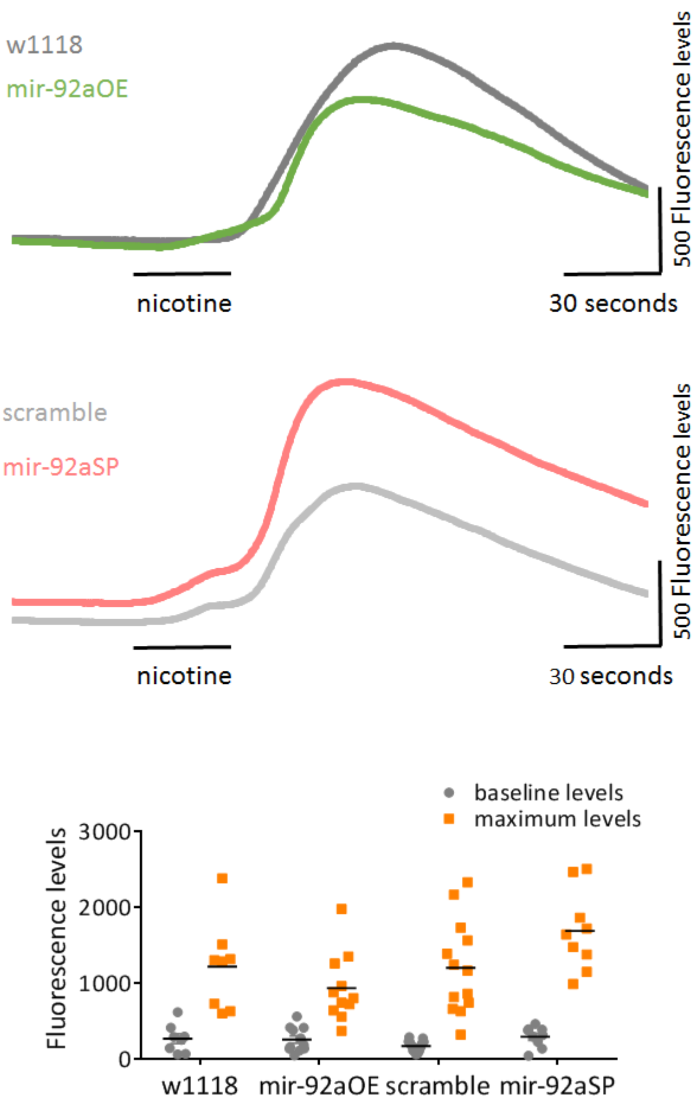

Supplementary Fig. 5 GCaMP6 live imaging of PDF neurons may show an altered responsiveness to nicotine in mir-92aOE and mir-92aSP flies. Flies expressing GCaMP6 in PDF cells (*PDF-GSG;UAS-GCaMP6f*) in addition to mir-92a manipulations (*UAS*-mir-92aOE or *UAS*-mir-92aSP) in an adult-specific manner were imaged for fluorescence level changes with 3x10^{-6} M nicotine perfusion after 30 seconds of baseline recording and the washed-out at 60 seconds. Flies were fed on food containing 0.2 mg/mL Mifepristone (Sigma-Aldrich) for 1 week and entrained for at least 3 LD cycles prior to the assay. Measurements for both scramble and mir-92aSP were performed between ZT18 – 22 (when endogenous mir-92a levels in PDF cells are high), and w1118 and mir-92aOE between ZT6 – 10 (when endogenous mir-92a levels in PDF cells are low). Average fluorescence levels of PDF cell bodies (l-LNvs) are plotted (top panels) and quantified (bottom panel). Each dot represents one brain. Bars represent the mean. Changes are statistically insignificant by two-way ANOVA.

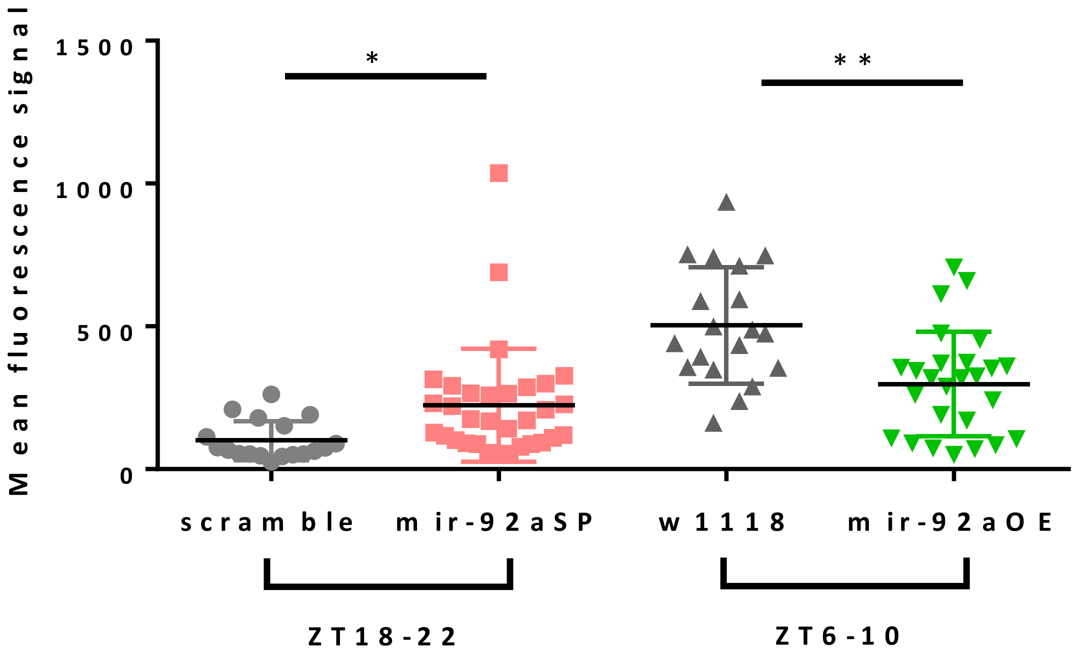

Supplementary Fig. 6 GCaMP6 baseline fluorescence levels in PDF cell projections are affected by manipulation of mir-92a in PDF cells.

Flies expressing GCaMP6 in PDF cells (*PDF-GAL4;UAS-GCaMP6f*) in addition to mir-92a manipulation (*UAS*-mir-92aOE or *UAS*-mir-92aSP) were imaged for baseline fluorescence levels. Fluorescence levels in PDF cell projections (s-LNvs) are plotted. Each dot represents an individual measurement. Measurements for both scramble and mir-92aSP were performed between ZT18 – 22 (when endogenous mir-92a levels in PDF cells are high), and w1118 and mir-92aOE between ZT6 – 10 (when endogenous mir-92a levels in PDF cells are low). Error bars represent ±SD, *$P < 0.05$, ** $P < 0.01$, two-tailed t-test.

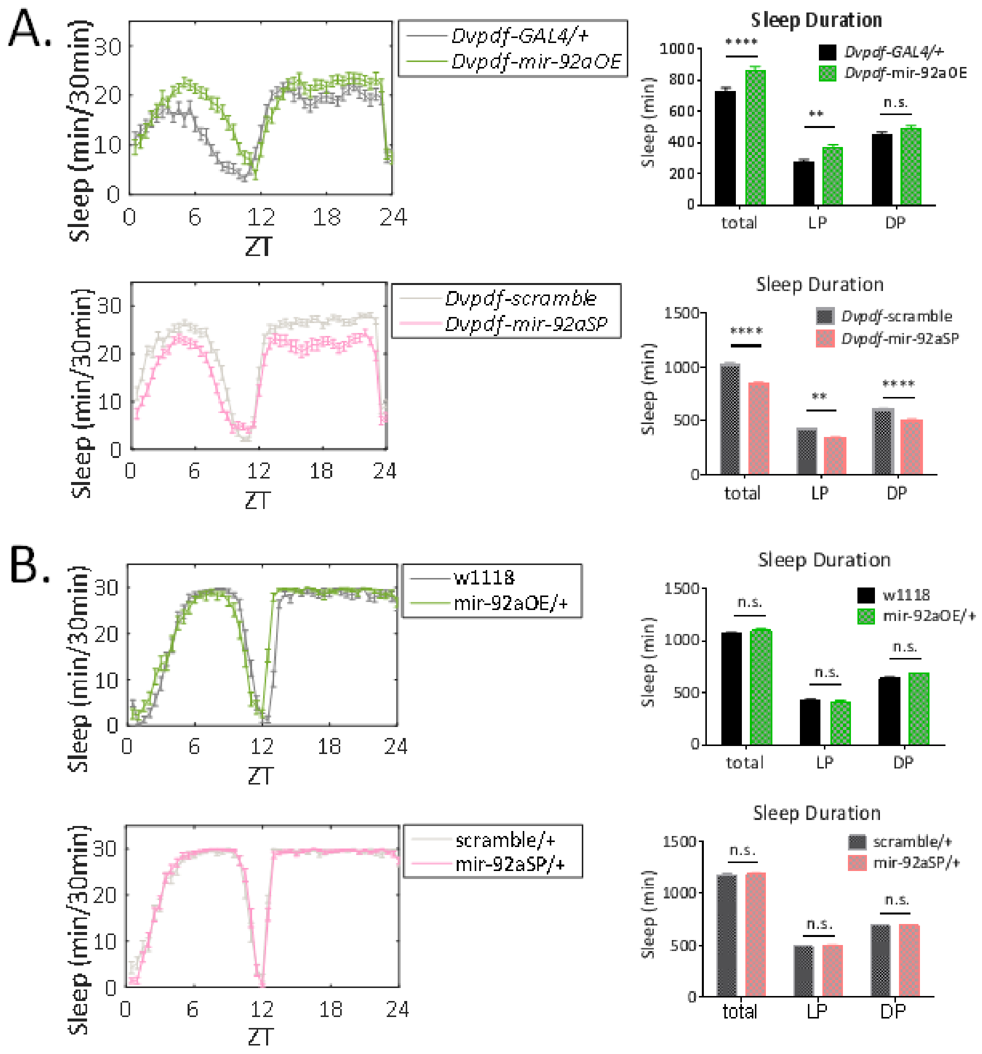

Supplementary Fig. 7 Sleep profiles of mir-92a mutant and control flies.

(A) Sleep profiles of female flies entrained under LD cycles. mir-92a levels were manipulated in wake-promoting neurons driven by *Dvpdf-GAL4*. (B) Sleep profiles from the control experiment with no GAL4 driver. Sleep duration is quantified to the right. N = 32. Error bars represent ±SEM, n.s. represents non-significant, **$P < 0.01$, **** $P < 0.0001$, two-way ANOVA.

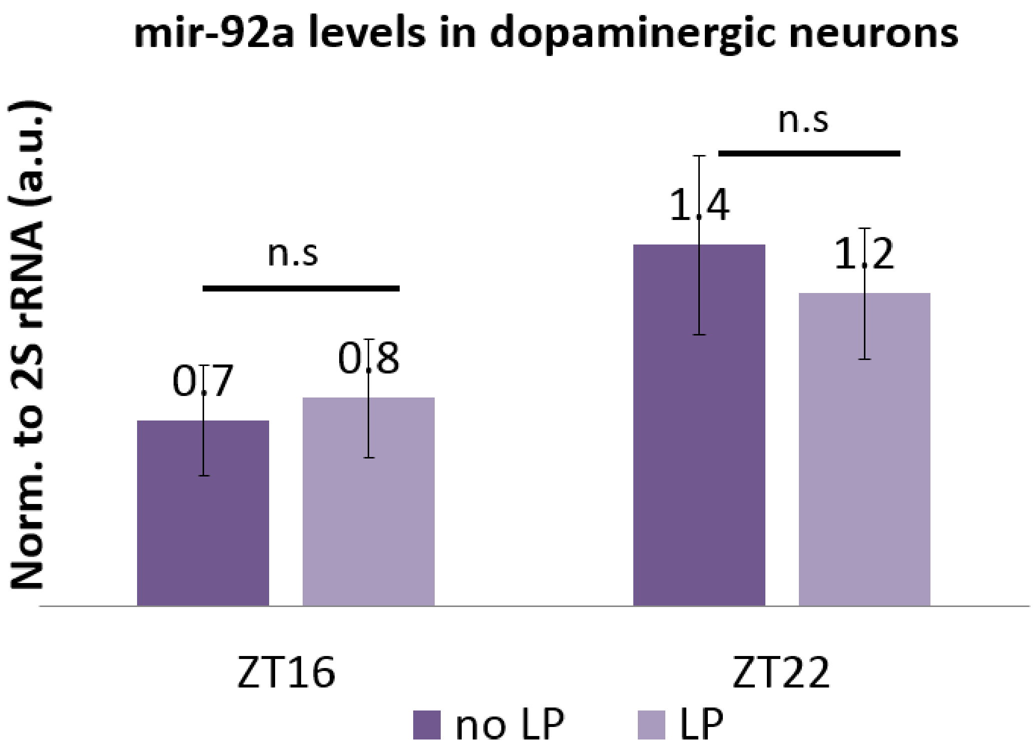

Supplementary Fig. 8 mir-92a expression levels in dopaminergic neurons of flies are not affected by light pulses.

RT-qPCR quantification of mir-92a levels in dopaminergic neurons. Flies were entrained for at least 3 days under LD cycles before exposure to a 10-min light pulse at either ZT15 or ZT21. Dopaminergic neurons were sorted at either ZT16 or ZT22 (50 min after the light pulse). No LP indicates no exposure to light, and LP indicates light pulse exposure. N = 3. Error bars represent ±SEM, n.s. represents non-significant, two-tailed t-test.

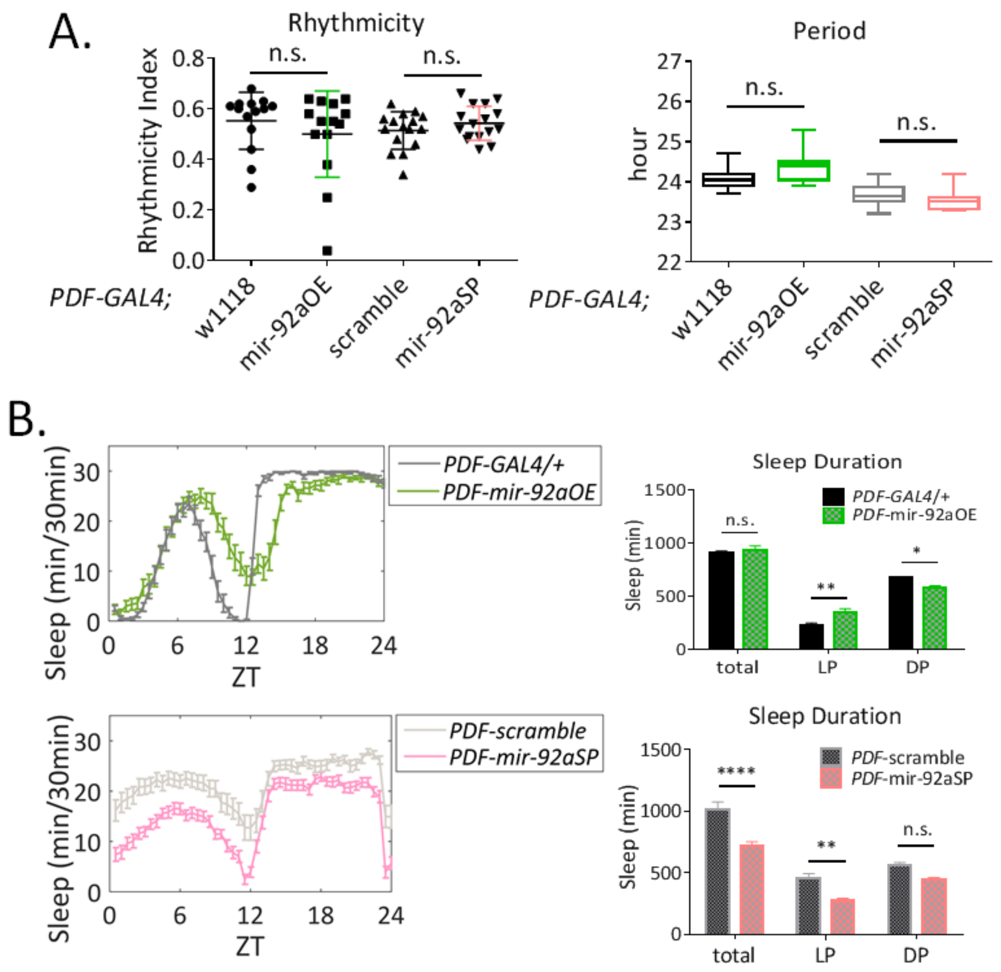

Supplementary Fig. 9 Sleep and circadian effects from manipulation of mir-92a levels in PDF cells.

(A) The left panel shows a scatter plot of rhythmicity index, and the right panel shows a box plot of period lengths. *PDF-GAL4* was crossed to w1118, *UAS*-mir-92aOE, *UAS*-scramble and *UAS*-mir-92aSP for comparison. Each dot indicates one fly. N = 16. Error bars represent ±SD, n.s. represents non-significant, one-way ANOVA.

(B) Sleep profiles of female flies of the same genotypes as in (A). Quantification of sleep duration is to the right. N = 16. Error bars represent ±SEM, n.s. represents non-significant, **P* < 0.05, ***P* < 0.01, **** *P* < 0.0001, two-way ANOVA.

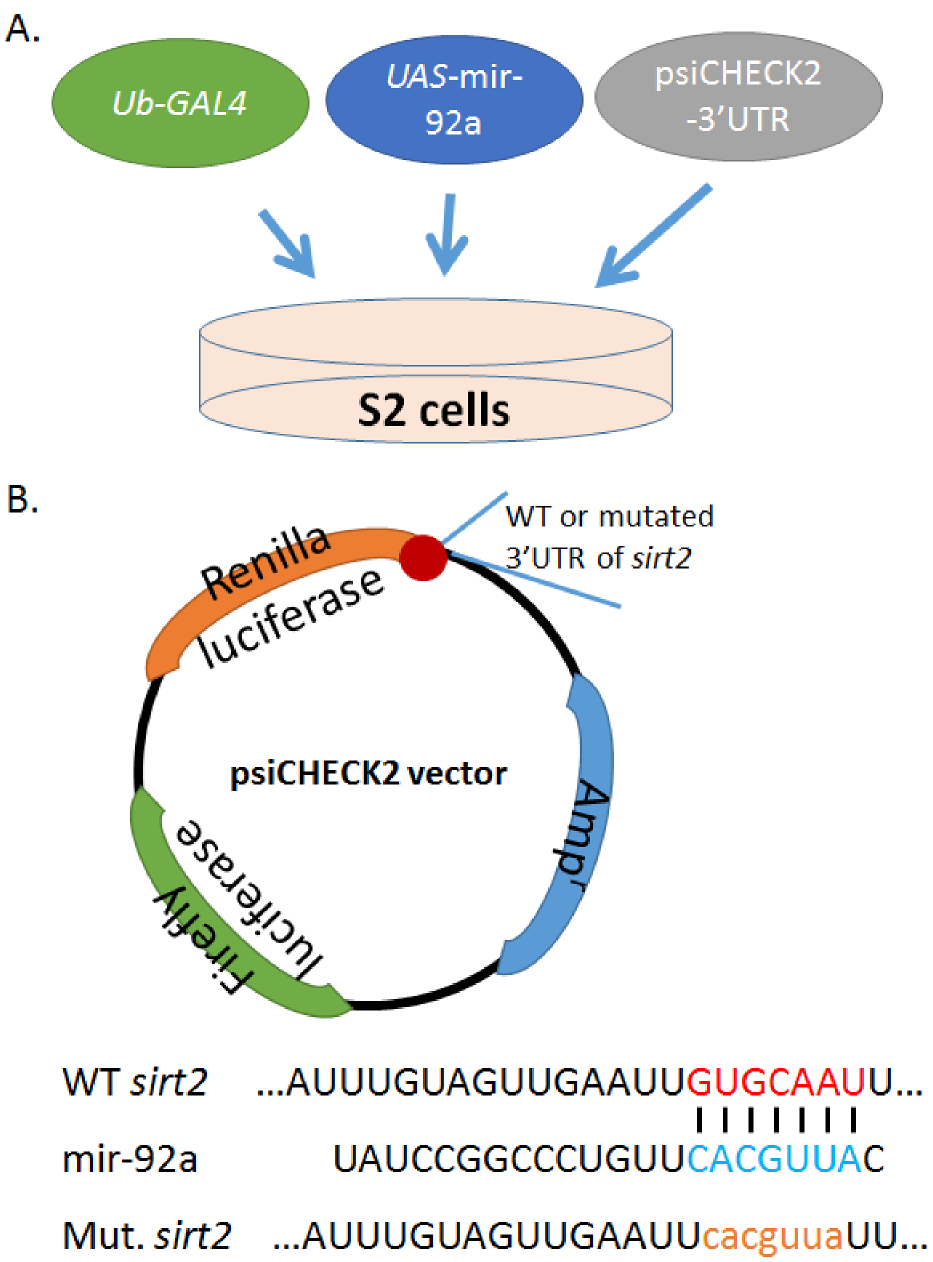

Supplementary Fig. 10 Scheme of S2 cell reporter assay.

(A) Three plasmids were co-transfected into S2 cells. *Ub-GAL4* and *UAS*-mir-92a co-transfection enabled expression of mir-92a in the cells, and psicheck2-3'UTR served as a reporter for the suppression assay.

(B) Plasmid map of psicheck2. The 3’UTR of *sirt2* was inserted downstream of *renilla*. WT *sirt2* 3’UTR shows base-paring with mir-92a, and the binding site in Mut. s*irt2* is mutated (brown). The plasmid map is adapted from Promega psiCHECK™-2 Vector.

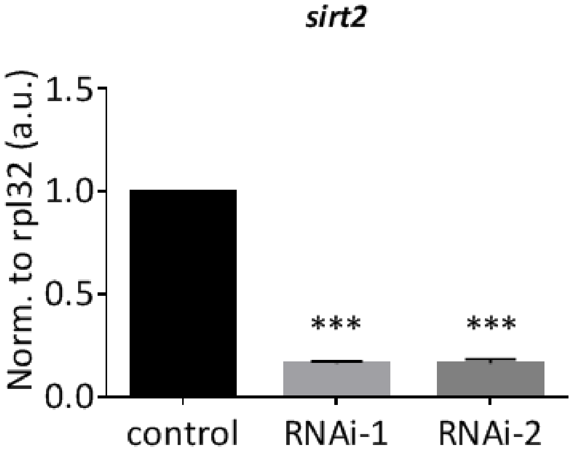

Supplementary Fig. 11 Quantification of *sirt2* RNAi efficiency.

Control (#36303, Bloomington stock center, WT background line for transgene injection) and two RNAi lines (RNAi-1: #32482; RNAi-2: #31363) were crossed to *Tubulin-GAL4* drivers for expression in whole flies. Total RNA was extracted from fly heads and *sirt2* mRNA levels were quantified with RT-qPCR with primers listed in Supplementary Table 2. N = 3. Error bars represent ±SEM, ****P* < 0.001, one-way ANOVA.

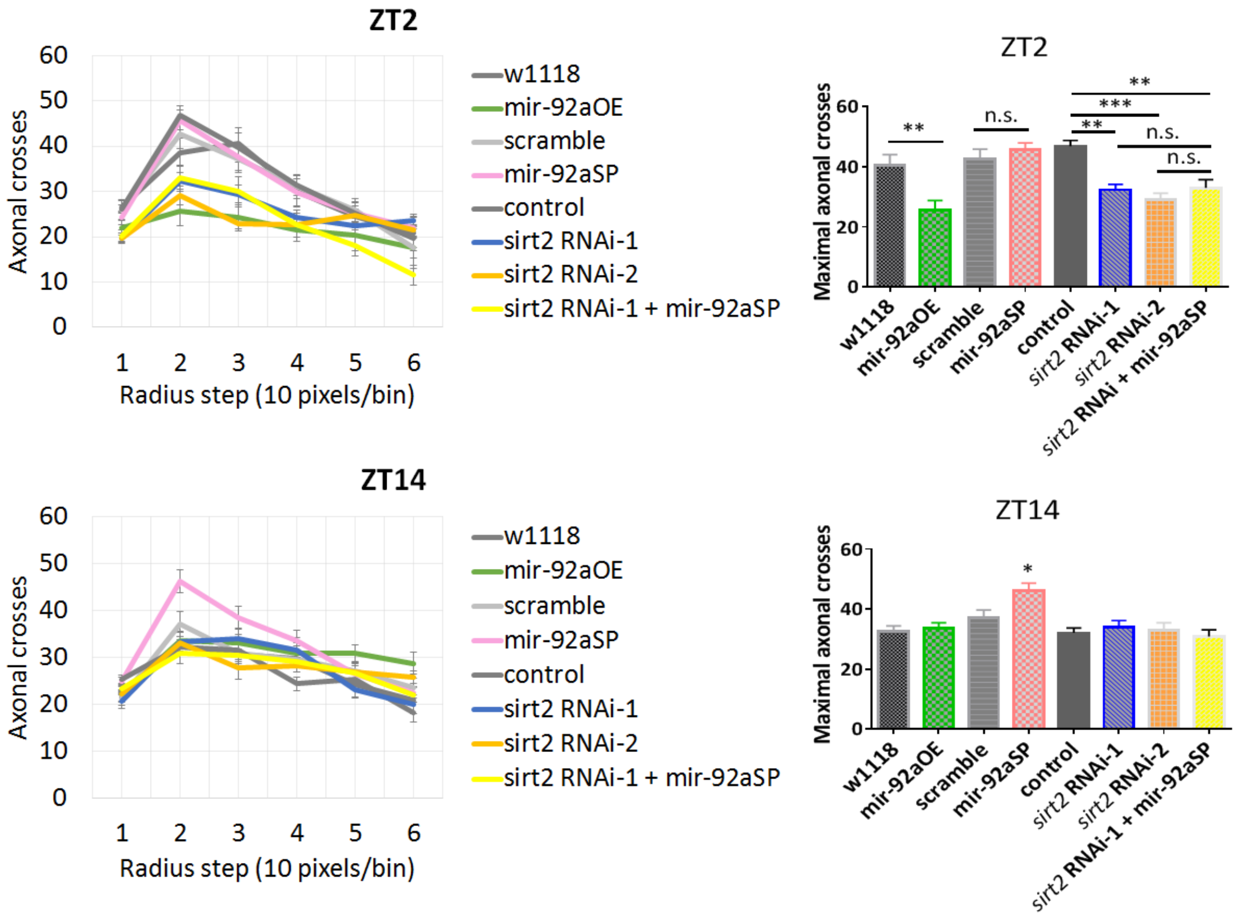

Supplementary Fig. 12 Quantification of PDF cell projection axonal crosses of the indicated genotypes.

Quantification with Sholl analysis was done as described above. All flies also express the *PDF-GAL4* driver. N = 14. Error bars represent ±SEM. n.s. represents non-significant, *$P < 0.05$, **$P < 0.01$, ***$P < 0.001$, one-way ANOVA.

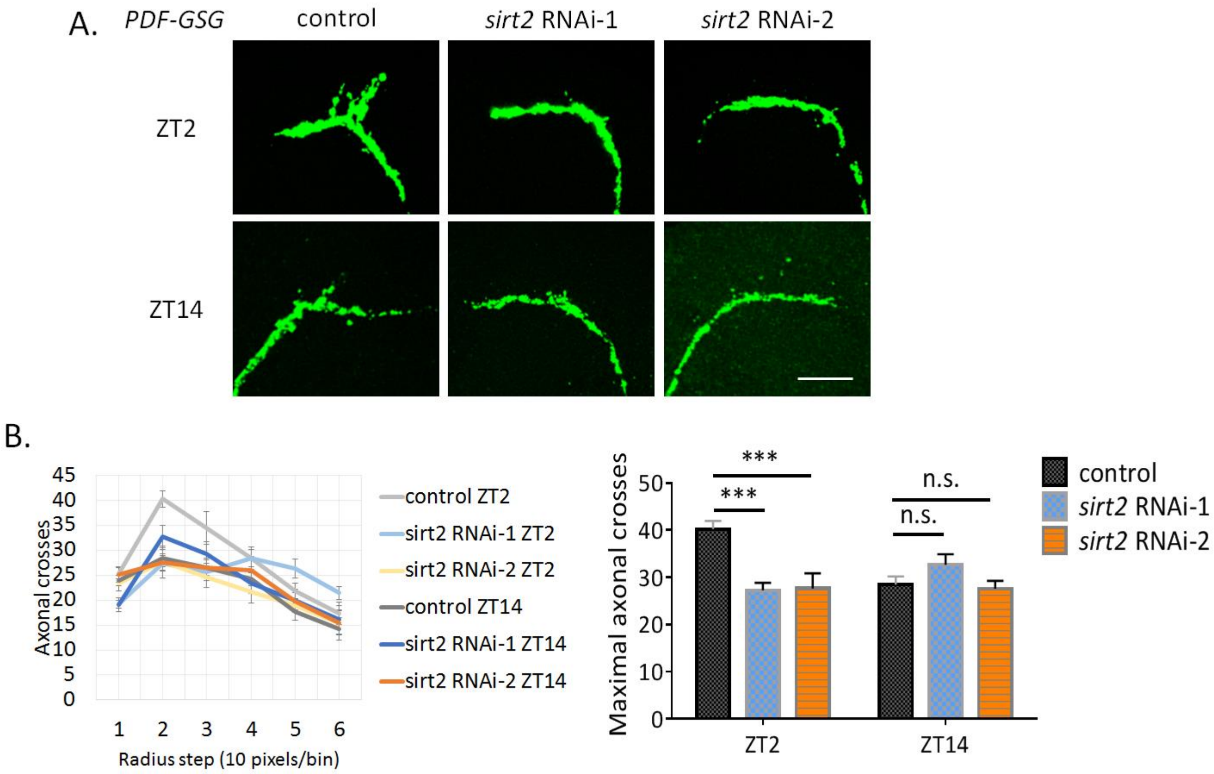

Supplementary Fig. 13 Adult-specific *sirt2* knockdown alters the PDF cell fasciculation phenotype.

Immunostaining of PDF cell projections with anti-PDF antibody at ZT2 or ZT14. The control is *PDF-GSG;UAS-mCD8::GFP/+* (with #36303 genetic background). *Sirt2* RNAi-1/2 is *PDF-GSG;UAS-mCD8::GFP;UAS-sirt2* RNAi-1/2. Flies were fed on food containing 0.2 mg/mL Mifepristone (Sigma-Aldrich) for 1 week and entrained for at least 3 LD cycles prior to the assay.

(A) Representative images of PDF cell projections of the indicated genotype at the indicated time. Scale bar equals 25 µm.

(B) Quantification was done with Sholl analysis. Statistics were done at the points with maximum axonal crosses. N = 14. Error bars represent ±SEM, n.s. indicates non-significant, ***$P < 0.001$, one-way ANOVA.

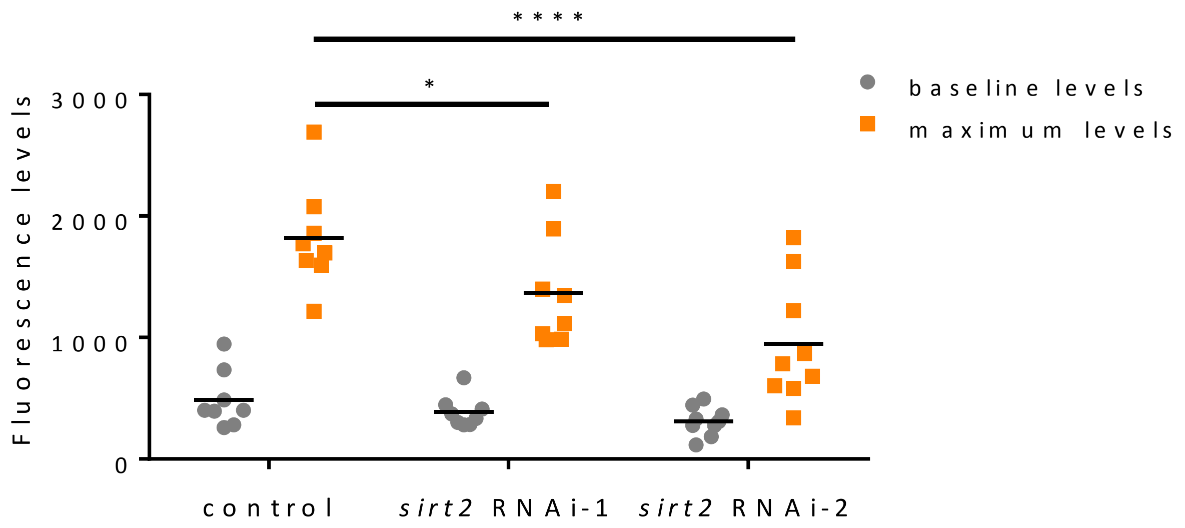

Supplementary Fig. 14 GCaMP6 live imaging of PDF neurons shows reduced responsiveness to nicotine with adult-specific *sirt2* knockdown.

Flies expressing GCaMP6 in PDF cells (*PDF-GSG;UAS-GCaMP6f*) in addition to RNAi against *sirt2* were imaged for fluorescence level changes with 3×10^{-6} M nicotine perfusion after 30 seconds of baseline recording and then a wash-out at 60 seconds. Flies were fed on food containing 0.2 mg/mL Mifepristone (Sigma-Aldrich) for 1 week and entrained for at least 3 LD cycles prior to the assay. Measurements were performed between ZT2 - 6. Average fluorescence levels in PDF cell bodies (l-LNvs) are quantified. Each dot represents one brain. Bars represent mean. *$P < 0.05$, ****$P < 0.0001$, two-way ANOVA.

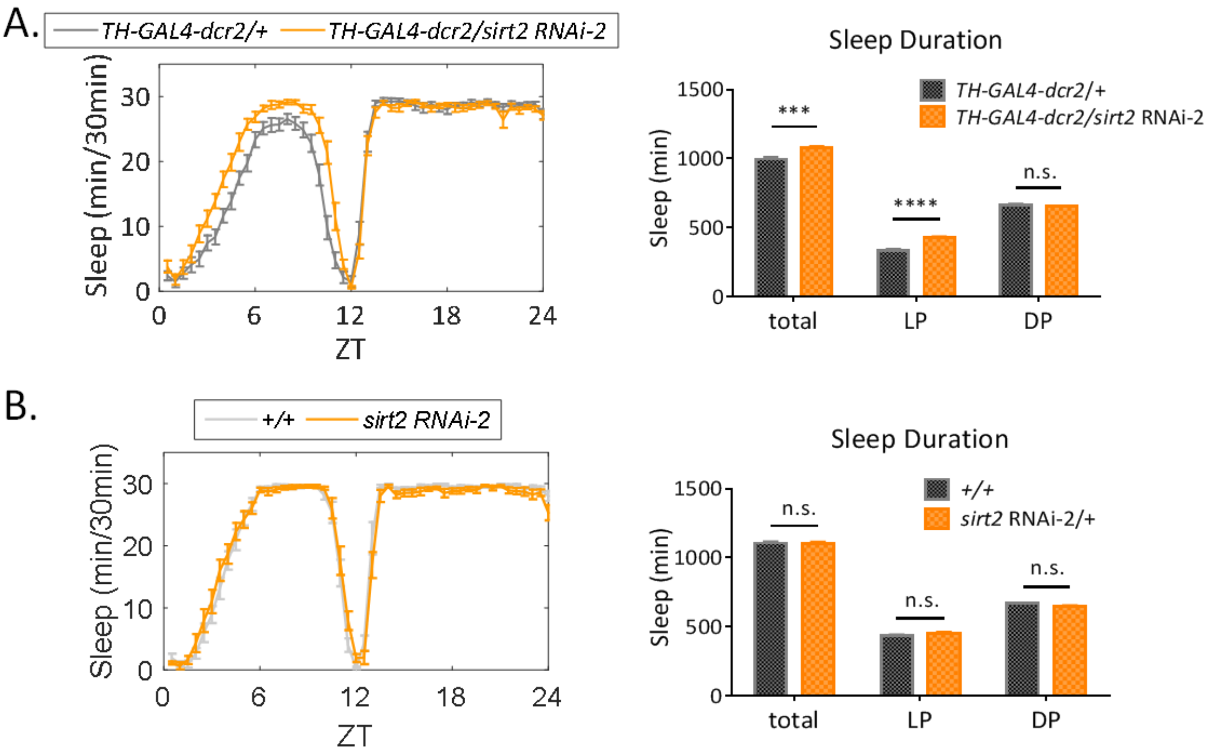

Supplementary Fig. 15 *sirt2* knockdown in dopaminergic neurons increases sleep duration.

(A) *sirt2* was knocked-down in dopaminergic neurons using *sirt2* RNAi-2 in combination with *TH-GAL4/UAS-dicer2* for higher knockdown efficiency. Sleep duration is quantified to the right. N = 16. Error bars represent ±SEM, n.s. represents non-significant, ****$P < 0.0001$, two-way ANOVA.

(B) A control experiment with no GAL4 driver. Sleep duration was quantified to the right. N = 16. Error bars represent ±SEM, n.s. represents non-significant, two-way ANOVA.

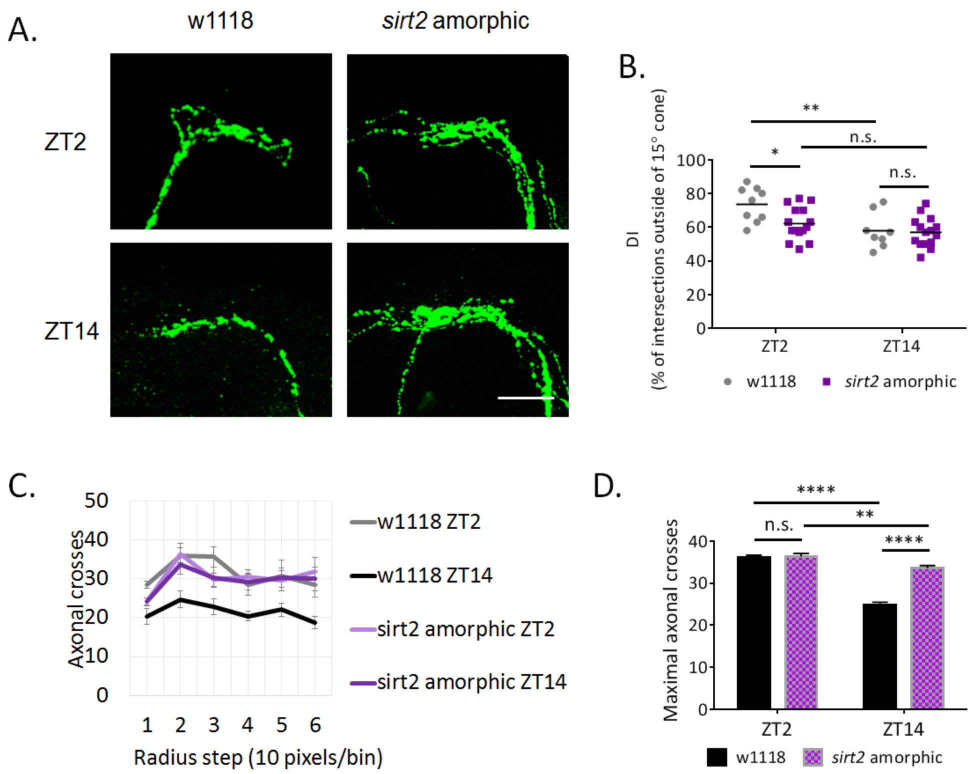

Supplementary Fig. 16 A *sirt2* amorphic mutant strain shows PDF projection abnormalities.

Immunostaining of PDF cell projections with anti-PDF antibody at ZT2 or ZT14. w1118 WT was used to compare with the *sirt2* mutant flies. Flies were entrained for at least 3 LD cycles prior to the assay.

(A) Representative images of PDF cell projections of the indicated genotypes at the indicated times. Scale bar equals 25 µm.

(B) The defasciculation index (DI) was calculated according to [31]. High DI indicates more defasciculation and lower DI indicates nire fasciculation. Each dot represents one projection. Bars indicate the mean. n.s. indicates non-significant, *$P < 0.05$, two-way ANOVA.

(C) Axonal crosses were quantified with Sholl analysis as described above.

(D) Statistics were done at the points with maximum axonal crosses. N = 8 - 15. Error bars represent ±SEM, n.s. indicates non-significant, **$P < 0.01$, ****$P < 0.0001$, two-way ANOVA.

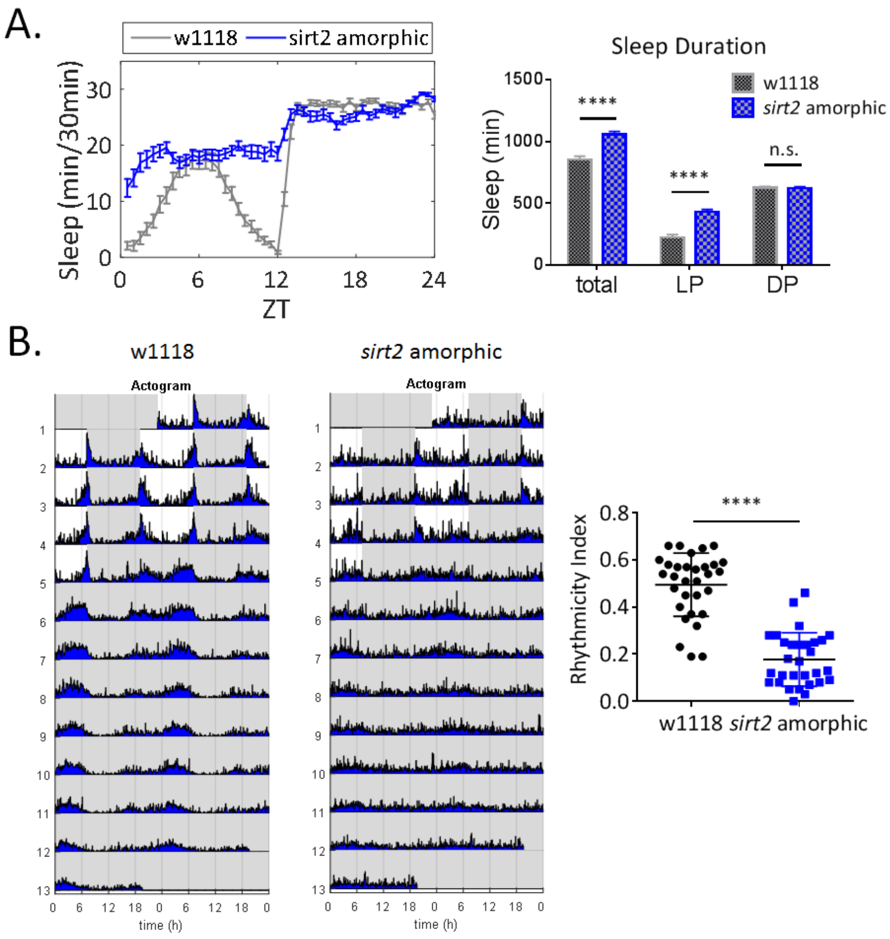

Supplementary Fig. 17 *sirt2* amorphic flies show increased sleep duration in LD as well as enhanced arrhythmicity in DD.

(A) Sleep profiles of female *sirt2* amorphic flies compared to w1118 flies under LD conditions. Quantification is to the right. N = 32. Error bars represent ±SEM, n.s. represents non-significant, ****$P < 0.0001$, two-way ANOVA.

(B) Actograms of male *sirt2* amorphic flies compared to w1118 flies during 3 days of LD cycles followed by 8 days of DD cycles. The actograms show the average activity of 32 flies. The white background indicates lights-on, and the grey background indicates lights-off. The rhythmicity index was calculated for individual flies. N = 32. Error bars represent ±SD, ****$P < 0.0001$, two-tailed t-test.

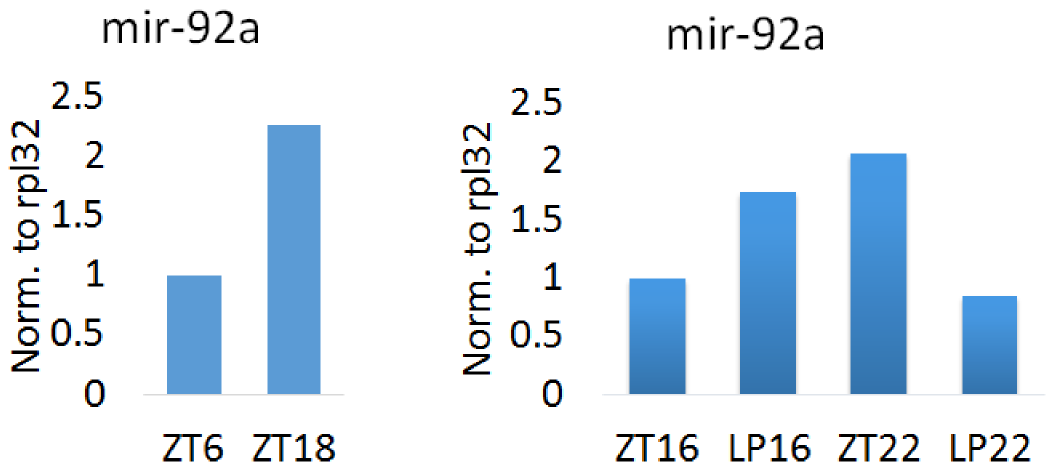

Supplementary Fig. 18 Validation of mir-92a expression in PDF cells using traditional stem-loop RT-qPCR.

Extracted RNA from sorted PDF cells was quantified. Shown to the left are PDF cell mir-92a levels at ZT6 and ZT18 from flies entrained under LD cycles. Shown to the right are PDF cell mir-92a levels from flies exposed to a 10-min light pulse at either ZT15 or ZT21 (same paradigm as Fig. 4A). ZT16/22 indicates mir-92a expression in controls with no exposure to light pulses. LP16/22 indicates mir-92a expression in flies with light pulse exposure. N = 2 biological replicates. Bar graphs show the average values of the replicates.

Supplementary Table 1 TRAP values of mir-92a target candidates with decreased IP/IN levels in mir-92a overexpression flies and increased levels in mir-92aSP flies

GENE NAMES	MIR-92AOE/W1118 (IP/IN)/(IP/IN)-1	MIR-92AOE/W1118 (IP/IN)/(IP/IN)-2	AVERAGE MIR-92AOE/W1118 (IP/IN)/(IP/IN)	MIR-92ASP/SCRAMBLE (IP/IN)/(IP/IN)-1	MIR-92ASP/SCRAMBLE (IP/IN)/(IP/IN)-2	AVERAGE MIR-92ASP/SCRAMBLE (IP/IN)/(IP/IN)
**CG30392**	0.29	0.56	0.42	1.64	1.88	1.76
**CG12024**	0.36	0.52	0.44	1.04	1.46	1.25
**CG1275**	0.58	0.55	0.56	1.59	2.20	1.90
**CG1275**	0.58	0.56	0.57	1.58	2.18	1.88
**CG2162**	0.73	0.63	0.68	1.16	1.53	1.34
**REGUCALCIN**	0.46	0.23	0.35	1.21	1.25	1.23
**CG4239**	0.46	0.55	0.50	2.51	1.50	2.00
**CG7990**	0.21	0.14	0.17	5.70	4.33	5.02
**CG7430**	0.77	0.68	0.72	1.42	1.21	1.31
**L(3)73AH**	0.17	0.60	0.39	1.36	2.07	1.71
**KAT60**	0.62	0.58	0.60	1.19	1.42	1.31
**RAB30**	0.00	0.93	0.46	7.85	1.44	4.64
**ARR1**	0.79	0.52	0.65	1.12	2.11	1.62
**DER-2**	0.74	0.68	0.71	1.43	1.47	1.45
**CG3534**	0.65	0.62	0.63	1.28	1.06	1.17
**CG14321**	0.14	0.67	0.41	1.60	3.28	2.44
**SIRT2**	0.40	0.47	0.43	1.84	1.13	1.49
**CG31038**	0.77	0.60	0.68	1.24	2.71	1.98
**HIL**	0.39	0.37	0.38	1.67	1.93	1.80
**CG11807**	0.07	0.35	0.21	1.97	1.93	1.95
**CG8323**	0.21	0.38	0.30	1.98	1.42	1.70
**ONECUT**	0.00	0.46	0.23	2.68	2.40	2.54
**LOLA**	0.00	0.49	0.24	1.13	4.32	2.73
**CG10737**	0.26	0.52	0.39	2.05	1.05	1.55
**PDK**	0.93	0.64	0.78	1.60	1.09	1.34
**ELAV**	0.67	0.54	0.60	2.68	1.52	2.10
**CG11807**	0.22	0.38	0.30	2.01	1.53	1.77
**POR**	0.92	1.00	0.96	3.46	5.65	4.56

Supplementary Table 2 Primer list

sirt2 3'UTR fwd	GCAGTAATTCTAGGCGATCGCACCCTAAGATTAGTTAGTAACATCCGTAGTTAATTTGTAGTTG
sirt2 3'UTR rv	AATGAAAATAAAGATATTTTATTGCGGCCAGCTTAGCCAGCAATGCCTGCGCTT
sirt2 mut 3'UTR fwd	CCGTAGTTAATTTGTAGTTGAATTcacgttaTTGTTTACATGTGGATTAC
sirt2 mut 3'UTR rv	GTAATCCACATGTAAACAAtaacgtgAATTCAACTACAAATTAACTACGG
Forward primer: Small RNA PCR Primer 2	AATGATACGGCGACCACCGACAGGTTCAGAGTTCTACAGTCCGA
Reverse primer	CAAGCAGAAGACGGCATACGAGATACATCGGTGACTGGAGTTATTGATGGTGCCTACAG
RT primer	ATTGATGGTGCCTACAG
3' linker (linker1)	rAppCTGTAGGCACCATCAAT/ddC/
5 'linker	GUUCAGAGUUCUACAGUCCGACGAUC
dme-mir-92a-3p fwd pr	ACACTCCAGCTGGGcattgcacttgtccc
2S fwd pr	ACACTCCAGCTGGGtacaaccctcaacca
dme-mir-184 fwd pr	ACACTCCAGCTGGGtggacggagaactga
dme-mir-999 fwd pr	ACACTCCAGCTGGGtgttaactgtaagac
dme-mir-981 fwd pr	ACACTCCAGCTGGGttcgttgtcgacgaa
dme-mir-210 fwd pr	ACACTCCAGCTGGGttgtgcgtgtgacag
dme-mir-276a fwd pr	ACACTCCAGCTGGGtaggaacttcatacc
sirt2 fwd	TTACCATGGGCGACATCGAA
sirt2 rv	GTCGTCACTGGACGAGGAAT
